# Purification and Characterization of a Novel Antiplatelet Peptide from *Deinagkistrodon acutus* Venom

**DOI:** 10.3390/toxins10080332

**Published:** 2018-08-16

**Authors:** Yi Kong, Qing Sun, Qi Zhao, Yaqiong Zhang

**Affiliations:** School of Life Science and Technology, China Pharmaceutical University, 24 Tong Jia Street, Nanjing 210009, China; qingsun@stu.cpu.edu.cn (Q.S.); qizhao@stu.cpu.edu.cn (Q.Z.); yaqiongzhang@stu.cpu.edu.cn (Y.Z.)

**Keywords:** *Deinagkistrodon acutus*, DAA-8, antiplatelet, PAR-1

## Abstract

Animal venoms are considered as one of the most important sources for drug development. *Deinagkistrodon acutus* is famous for its toxicity to the human hematological system and envenomed patients develop a coagulation disorder with the symptoms of hemorrhage and microthrombi formation. The purpose of this study was to separate antiplatelet peptides from *D. acutus* venom using a combination of an ultrafiltration technique and reversed-phase high performance liquid chromatography (HPLC), which was guided by monitoring antiplatelet aggregation bioactivity. A novel octa-peptide named DAA-8 was found. This peptide inhibited protease-activated receptor1 (PAR-1) agonist (SFLLRN-NH_2_) induced platelet aggregation and it also inhibited platelet aggregation induced by thrombin, ADP, and collagen. Furthermore, DAA-8 showed significant antithrombotic activity and resulted in a slightly increased bleeding risk in vivo. This is the first report of a peptide derived from snake venom, which inhibited PAR-1 agonist-induced platelet aggregation. This peptide may provide a template to design a new PAR-1 inhibitor.

## 1. Introduction

Thrombosis plays an important role in the pathogenesis of many cardiovascular or cerebrovascular diseases, such as acute coronary syndrome, transient ischemic attack, atherosclerosis, and stroke [1,2]. Platelets play a pivotal role during the formation of thrombus. When blood vessels are injured, platelets adhere to the exposed subendothelial matrix and are then activated, releasing the agonists ADP, thromboxane A2, thrombin, and epinephrine. Finally, many platelets aggregate at the injured vascular site and form plugs [3,4,5]. Antiplatelet medicines have been used in clinics as a tool for preventing thrombotic diseases. However, the present drugs result in increased hemorrhagic risk [6]. Research to find more effective and safer antiplatelet agents is needed.

Animal venoms are considered as one of the most important sources for drug development [7]. *D. acutus* (Chinese moccasin or five-pace snake) is considered as the monovalent genus *Deiagkistrodon* previously classified into one genus *Agkistrodon*, which lives mainly in southeast China, North Vietnam, Laos, and Taiwan [8]. *D. acutus* envenomation causes hemocoagulation disorder, which causes the symptoms of hemorrhage and microthrombi formation. In several severe cases, this can lead to death [9]. Many studies indicated that in particular, this venom contains activating or inhibiting factors of the plasmatic coagulation system, thrombin-like enzymes for fibrinogen conversion, platelet aggregation inhibitors, and even platelet activators, etc. [10,11,12].

The purpose of the present study was to separate antiplatelet peptides from *D. acutus* venom. A novel octa-peptide DAA-8 was found. This peptide inhibited SFLLRN-NH_2_ (specific for PAR-1)-, thrombin-, ADP-, and collagen-induced platelet aggregation in vitro. It also decreased thrombotic formation in a rat arterio-venous shunt thrombosis model in vivo.

## 2. Results

### 2.1. Purification of Antiplatelet Aggregation Peptides from D. acutus Venom

**Figure 1:**
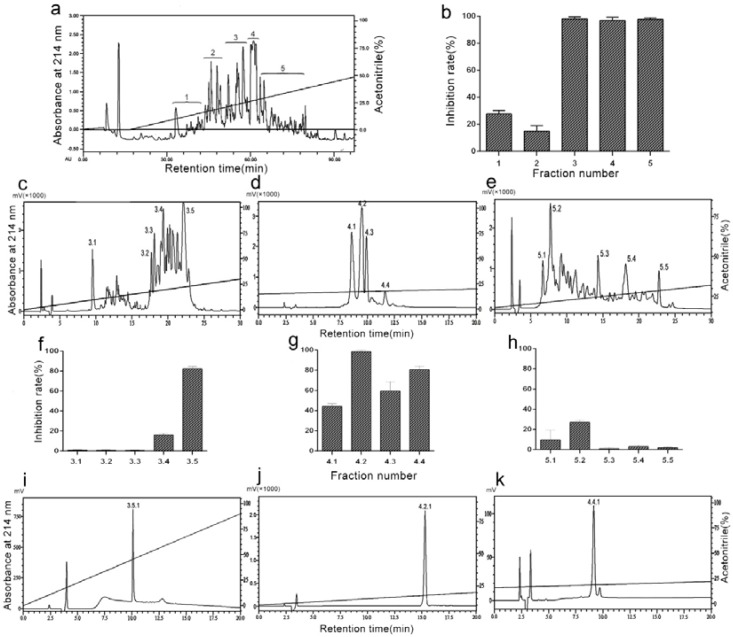
Bioassay-directed chromatographic separation of anti-platelet aggregation peptide. (**a**) The lyophilized percolate (*D. acutus* venom) was separated on a semi-preparative RP-HPLC column (Hedera ODS-2; 250 × 20 mm, 5 µm) in the BioLogic Duoflow system (Bio-Rad, Redmond, WA, USA) using acetonitrile as the organic modifier and trifluoroacetic acid (TFA) as the ion-pairing reagent. (**b**) Inhibitory activities of fractions 1–5 against thrombin-induced platelet aggregation. Platelet aggregation inhibition rate (%) = [(X−Y)/X]×100, where X represents the maximum aggregation rate of saline-treated gel-filtered platelet and Y represents the maximum aggregation rate of sample-treated gel-filtered platelet. (**c**–**e**) The Fractions 3, 4, and 5 were separated on an analytical RP-HPLC column (Diamonsill C18; 4.6 × 250 mm, 5 µm). (**f**–**h**) Inhibitory activities of fractions 3.1–3.5, 4.1–4.4, and 5.1–5.5 against thrombin-induced platelet aggregation. (**i**–**k**) The Fractions 3.5, 4.2, and 4.4 were separated on an analytical RP-HPLC column (Diamonsill C18; 4.6 × 250 mm, 5 µm). Data are presented as means ± SD (*n* = 3).

The purification procedure consisted of a combination of an ultrafiltration technique and a reversed-phase chromatography, which was guided by monitoring thrombin induced-platelet aggregation. The venom was dissolved in 50mM Tris-HCl (0.9% NaCl, pH 8.5) and was applied to an ultrafiltration tube with a molecular weight cut-off value of 10KD. The components passing through the ultrafiltration membrane were collected, before being further purified by RP-HPLC on a Hedera ODS-2 column (20 × 250 mm) equilibrated with 10% acetonitrile containing 0.1% trifluoroacetic acid (TFA). The elution profile obtained on a linear gradient of 0–50% acetonitrile containing 0.1% TFA included five fractions, which were designated as 1–5 (Figure 1a). Each fraction was collected and independently tested for antiplatelet aggregation activity, with the Fractions 3, 4, and 5 displaying strong inhibitory action (Figure 1b). They were further isolated by an analytical C18 column (4.6 × 250 mm) using a Shimadzu LC-20AT HPLC system (Figure 1c–e). Each fraction was collected and tested for antiplatelet aggregation activity (Figure 1f–h). Fractions 3.5, 4.2, and 4.4 had high activity and were further isolated by C18 column (4.6 × 250 mm) under different elution conditions (Figure 1i–k). We collected the Fractions 3.5.1 (Figure 1i), 4.2.1 (Figure 1j), and 4.4.1 (Figure 1k). Furthermore, we tested their antiplatelet bioactivities (data not shown) and these three fractions were independently sequenced by Edman protein sequencer. The sequence of Fraction 4.4.1 was IIWTEEDK, which was termed DAA-8. The sequences of Fractions 3.5.1 and 4.2.1 were not revealed because their N-terminals were blocked.

### 2.2. DAA-8 Inhibited Platelet Aggregation in Vitro

**Figure 2:**
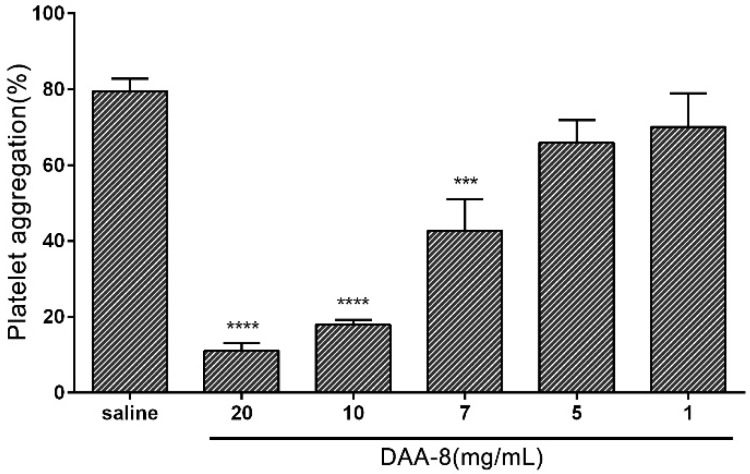
Effects of DAA-8 on platelet aggregation induced by thrombin. Gel-filtered platelets were preincubated for 5 min with different concentrations of DAA-8 (1, 5, 7, 10, and 20 mg/mL) or vehicle controls (saline). Platelet aggregation was initiated with thrombin (0.1 U/mL). Data are presented as means ± SD of three independent experiments. **** *p* < 0.0001 versus vehicle and *** *p* < 0.001 versus vehicle, analyzed by one-way ANOVA, which was followed by Dunnett’s multiple comparisons test.

**Figure 3:**
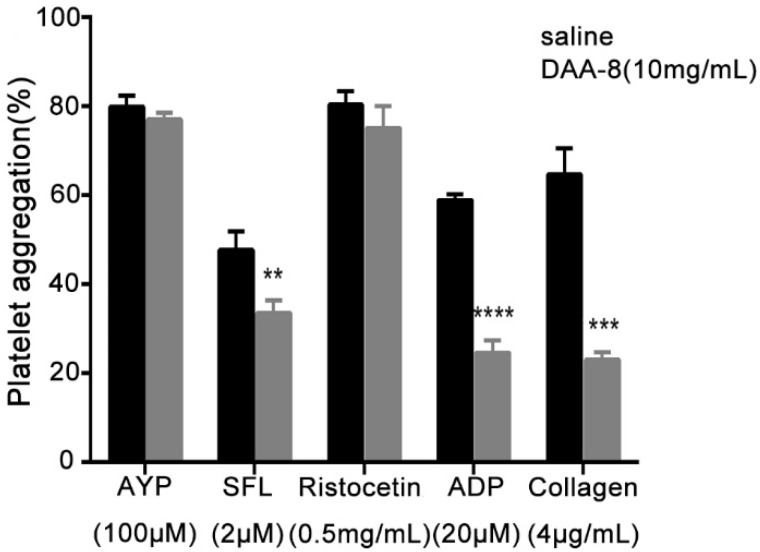
DAA-8 inhibited SFL-, ADP- and collagen-induced human platelet aggregation in vitro. Gel-filtered platelets were preincubated for 5 min with DAA-8 (10 mg/mL) or vehicle. Platelet aggregation was initiated with AYP (100 µM), SFL (2 µM), ristocetin (0.5 mg/mL), ADP (20 µM) or collagen (4 μg/mL). Data are means ± SD (*n* = 3), **** *p* < 0.0001, *** *p* < 0.001, and ** *p* < 0.01 versus vehicle, which was analyzed by Student's *t*-test using two-sample comparison.

To study the effect of DAA-8 on platelet aggregation, we used gel-filtered human platelets induced with various agonists as an in vitro model. The results showed that DAA-8 selectively inhibited platelet aggregation, which was induced by different agonists. As shown in Figure 2, DAA-8 inhibited thrombin (0.1 U/mL)-induced platelet aggregation in a dose-dependent manner, with IC_50_ value of 7.29 mM (95% CI, 6.28–8.29 mM). Thrombin has three receptors: protease-activated receptor 1(PAR-1), protease-activated receptor 4 (PAR-4) and GPIb. The inhibitory effect of DAA-8 on platelet aggregation that was induced by three specific agonists (SFLLRN-NH_2_ for PAR-1, AYPGKF-NH_2_ for PAR-4 and ristocetin for GPIb) were tested independently (Figure 3). The peptide inhibited SFLLRN-NH_2_ (2 µM)-induced platelet aggregation by 29.44 ± 5.80% (mean ± SD, *n* = 3), but it exerted very little inhibitory effects on AYPGKF-NH_2_ (100 µM) and ristocetin (0.5 mg/mL)-induced platelet aggregation. At even 10 mg/mL, the peptide showed only 3.58 ± 1.75% and 6.55 ± 6.13% inhibitory rates (mean ± SD, *n* = 3) against AYPGKF-NH_2_ (100 µM) and ristocetin (0.5 mg/mL)-induced platelet aggregation, respectively. In addition, DAA-8 inhibited ADP (20 µM)- and collagen (4 µg/mL)-induced platelet aggregation with inhibitory rates of 58.26 ± 4.72% and 64.35 ± 2.62% (mean ± SD, *n* = 3), respectively.

### 2.3. DAA-8 Inhibited Arterio-Venous Shunt Thrombosis in Rat

**Figure 4:**
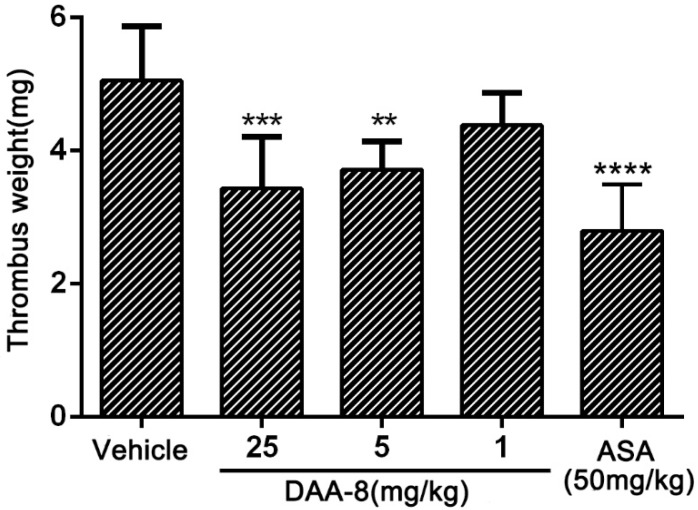
DAA-8 inhibited arterio-venous shunt thrombosis in rats in vivo. Sprague Dawley rats were weighed and anesthetized by an intraperitoneal injection of 10% chloral hydrate (5 mL/kg). An arterio-venous shunt tube (12 cm, containing single silk thread with a length of 10 cm) was installed between the right carotid artery and left jugular vein of each rat. After the blood circulated through the shunt tube for 20 min, the silk thread carrying thrombus was pulled out from the shunt tube. The dry weight of thread was measured 40 min later at 60 °C. The weights of the formed thrombi were determined by subtracting the pre-experimental weights of the dry 10-cm threads. Data are mean ± SD, ** *p* < 0.01, *** *p* < 0.001, and **** *p* < 0.0001 vs. vehicle, *n* = 6, analyzed by one-way ANOVA, which was followed by Dunnett’s multiple comparisons test.

The effect of DAA-8 on thrombus formation was evaluated in the rat arterio-venous shunt thrombosis model. As illustrated in Figure 4, DAA-8 inhibited thrombosis formation in a dose-dependent manner. After administration of DAA-8 (i.v.) at 1, 5, and 25 mg/kg, the weight of the thrombus was reduced by 13.20 ± 9.65%, 26.40 ± 8.44%, and 32.01 ± 15.32% (mean ± SD, *n* = 6), respectively.

### 2.4. DAA-8 Exhibited Low Bleeding Risk in Mice

**Figure 5:**
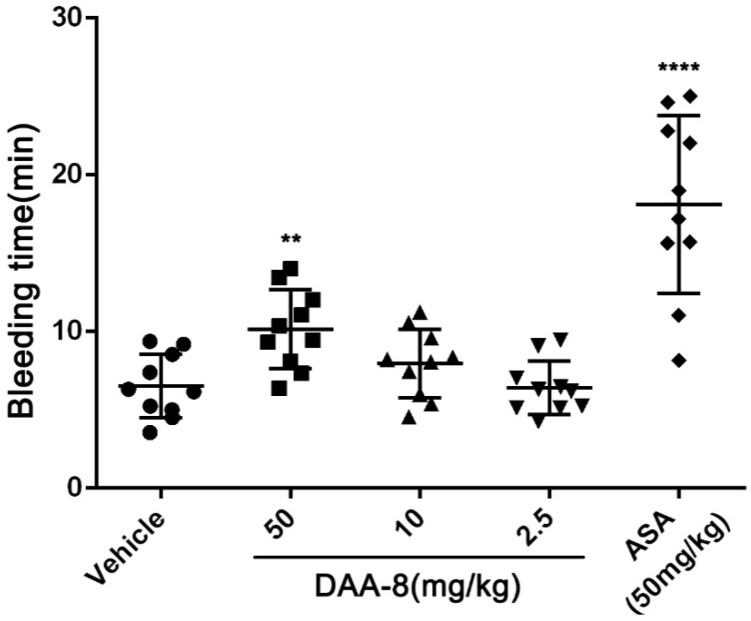
DAA-8 exhibited low bleeding risk in mice. Fifteen min after the administration of DAA-8 (2.5, 10, and 50 mg/kg), aspirin and vehicle, mice were anesthetized with intraperitoneal 5% chloral hydrate (6 mL/kg). After this, a 3-mm length of mice tail tip was cut, before the remaining tail was immersed immediately into saline at 37 °C. The accumulated bleeding time (including periods of re-bleeding) was recorded within a 20-min period. Data are presented as means ± SD (*n* = 10). **p < 0.01 and ****p < 0.0001 compared with vehicle, analyzed by one-way ANOVA, which was followed by Dunnett’s multiple comparisons test.

To evaluate the bleeding risk of DAA-8, we tested the bleeding time of DAA-8-treated mice using a mice tail cutting assay at DAA-8 concentrations of 2.5, 10, and 50 mg/kg, which represents twice the doses for the anti-thrombotic studies. Saline-treated mice served as a negative control and aspirin-treated mice as a positive control. As shown in Figure 5, prolonged bleeding time was observed at a dose of 50 mg/kg of DAA-8 (10.15 ± 2.53 min, mean ± SD, *n* = 10). At doses of 2.5 and 10 mg/kg, the efficient doses that were required to prevent thrombus formation in mice, DAA-8 did not significantly prolong the bleeding time (6.42 ± 1.71 min and 7.95 ± 2.18 min, respectively; mean ± SD, *n* = 10) compared with the vehicle group (6.53 ± 2.03 min; mean ± SD, *n* = 10). This suggests that DAA-8 results in low bleeding risk. Aspirin significantly prolongs the bleeding time.

## 3. Discussion

Snake venom is a potential source for the discovery of bioactive substances [13]. In the present study, a novel octa-peptide with a molecular weight of 1032.25Da (Figure S1) and a sequence of IIWTEEDK named DAA-8 was isolated from the venom of *D. acutus* using a combination of an ultrafiltration technique and a reversed-phase HPLC. DAA-8 inhibited the agonist peptide SFLLRN-NH_2_ (specific for PAR-1)-induced platelet aggregation and this also inhibited platelet aggregation induced by thrombin, ADP, and collagen. DAA-8 showed significant antithrombotic activity and slightly increases the bleeding risk in vivo.

Many platelet aggregation inhibitors derived from snake venom have been reported. Rhodocetin from *Calloselasma rhodostoma* [14,15] and Flavocetin-A from *Trimeresurus flavoviridis* [16,17] inhibited platelet aggregation by targeting α2β1 integrin, while kistomin from *C. rhodostoma* inhibited CVX (specific for GPVI)-induced platelet aggregation at a dose of 1.2 µM [18]. Furthermore, Cc-Lec (34,271.59 Da) from *Cerastescerastes* inhibited platelet aggregation induced by ADP, arachidonic acid, or fibrinogen at a minimal dose of 10 µM [19].

DAA-8 is different from the above-mentioned inhibitors as it not only inhibited collagen and ADP-induced platelet aggregation but also interestingly inhibited PAR1 agonist (SFLLRN-NH2)-induced platelet aggregation. PAR-1, which is a seven transmembrane G-protein-coupled receptor, is one of three thrombin receptors (PAR-1, PAR-4, and GPIb) on the surface of platelets [20,21,22]. The inhibition of PAR1 can potently decrease platelet aggregation. PAR1 inhibitory peptides were seldom found in animal venoms. One reported platelet aggregation inhibitory peptide targeting PAR-1 is a fragment (Trp-Ile-Arg-Arg-Pro-Phe-Phe-Pro-Phe) of αB-crystallin [23]. The study of PAR1 antagonist mainly focuses on natural or synthesized small molecular chemical compounds. Vorapaxar, which is a PAR-1 inhibitor, has been approved by FDA as an antithrombotic drug but the clinic data showed that it can lead to brain bleeding [24,25]. Thus, the development of novel agents is necessary. DAA-8 exhibited a mild antithrombotic effect in an arterio-venous shunt model in rats and low bleeding risk in a tail-cutting mouse model compared to a positive control aspirin at 50 mg/kg. The usual adult dose of aspirin for ischemic stroke or angina pectoris prophylaxis is 50–325 mg once a day. According to the animal-to-human dose translation rule, 50 mg/kg aspirin in mice (20 g) can be translated to a dose of 388 mg in humans (70 kg). Thus, 50 mg/kg aspirin in mice roughly represents a therapeutic dose in humans. This indicates that the DAA-8 possesses an advantage of a high benefit/risk ratio. DAA-8 as a PAR-1 inhibitor may provide a template to design new PAR-1 inhibitor. However, the weak potency of this peptide should be improved.

We searched the sequence of DAA-8 using BLAST and found that DAA-8 is a fragment of acurhagin (in position aa249–256), which originates from *A. acutus* venom. It was reported that acurhagin dose-dependently inhibited platelet aggregation induced by collagen [26]. DAA-8 also exist in other proteins derived from *A. acutus*, such as a metalloproteinase Aahiv (aa56–63) [27] and a fibrinolytic enzyme F IIa (aa70–77) [28,29].

## 4. Conclusions

DAA-8 is a novel antiplatelet peptide isolated from *D. acutus* venom.DAA-8 inhibited SFLLRN-NH_2_, collagen, and ADP-induced platelet aggregation, while it also showed strong antithrombotic activity. To the best of our knowledge, this is the first PAR-1 antagonist peptide derived from *D. acutus* venom. The mechanism of DAA-8 needs to be further investigated.

## 5. Materials and Methods

### 5.1. Materials and Reagents

Lyophilized *D. acutus* venom was obtained from Jingdezhen Chen Feng special wildlife technology development Co. Ltd (Jingdezhen, Jiangxi province, China) and stored at −20 °C. Adenosine diphosphate (ADP), collagen and aspirin were purchased from Sigma Chemical Co. (St. Louis, MO, USA); human thrombin was purchased from Hyphen-Biomed (Neuville Sur Oise, France); ristocetin was purchased from American Biochemical and Pharmaceuticals (ABP Ltd., Marlton, NJ, USA); SFLLRN-NH_2_ and AYPGKF-NH_2_ were purchased from Ningbo Kangbei biochemical Co. Ltd. (Ningbo, Zhejiang province, China). All other chemicals used in this study were of analytical grade.

### 5.2. Animals and Human Samples

Adult institute of cancer research (ICR) mice and Sprague Dawley rats were obtained from Nanjing Qinglongshan Animal Centre (Nanjing, Jiangsu province, China). All animals were housed under controlled temperature (21–25 °C) and light (12h light, 12h dark), with ad libitum access to food and water for one week before experiments. All experiments were performed according to the guidelines and the regulations of the Ethical Committee of China Pharmaceutical University (CPU2016-S07, 5 March 2016). Human blood was obtained from healthy donors with informed consent according to the Declaration of Helsinki.

### 5.3. Purification of DAA-8

Two grams of crude venom was dissolved in 350 mL of 50 mM Tris-HCl buffer (0.9% NaCl, pH 8.5) and kept at 4 °C for 24 h. The solution was separated by centrifugation at 3500× g for 20 min and subjected to an ultrafiltration membrane with a cut-off molecular weight of 10,000 Da. The percolates were pooled and lyophilized. The lyophilized power was dissolved and loaded onto a semi-preparative RP-HPLC column (Hedera ODS-2; 250 × 20 mm, 5 µm) in the BioLogic Duoflow system (Bio-Rad, Redmond, WA, USA) using acetonitrile as the organic modifier and trifluoroacetic acid (TFA) as the ion-pairing reagent. Eluent A consisted of 0.1% TFA in 5% acetonitrile/H_2_O (*v/v*), while eluent B consisted of 0.1% TFA in 95% acetonitrile/H_2_O (*v/v*). The linear gradient elution conditions were conducted using an isocratic step for 0–100 mL with eluent A and a subsequent 500 mL linear gradient of 0–50% eluent B at a flow rate of 6.0 mL/min. The UV absorbance was monitored at 214 nm. The Fractions 3, 4, and 5 with high antiplatelet aggregation activity were further separated independently by analytical RP-HPLC columns (Diamonsill C18; 4.6 × 250 mm, 5 µm) in Shimadzu LC-20AT HPLC system using a linear acetonitrile gradient at a flow rate of 1.0 mL/min. A gradient elution was carried out according to the following processes: 0–30 min, 0–30% B for Fraction 3; 0–20 min, 15–20% B for Fraction 4; and 0–30 min, 18–32% B for Fraction 5. Eluates were monitored at 214 nm. Each fraction was evaluated for antiplatelet aggregation activity. The Fractions 3.5, 4.2, and 4.4, which displays strong antiplatelet aggregation activity, were further separated independently by an analytical RP-HPLC column (Diamonsill C18; 4.6 × 250 mm, 5 µm) using a linear acetonitrile gradient at a flow rate of 1.0 mL/min. A gradient elution was carried out according to the following processes: 0–20 min, 13–18% B for Fraction 3.5; 0–20 min, 10–20% B for Fraction 4.2; and 0–20 min, 16–20% B for Fraction 4.4. Each fraction was evaluated for antiplatelet aggregation activity. Fractions 3.5.1, 4.2.1 and 4.4.1—which display strong antiplatelet aggregation activity—were collected and lyophilized. The purity of the samples was checked on the analytical C18 RP-HPLC column using Shimadzu LC-20AT system. The purified peptide was sequenced by Edman degradation using protein sequencer, model PPSQ-31A (Shimadzu, Japan).

### 5.4. Platelet Preparation

Blood was drawn from healthy volunteers into the siliconized vacutainers, which contain a 1:5 volume anticoagulant citrate dextrose (ACD). To obtain Platelet-rich plasma (PRP), the blood was centrifuged at 168× g for 5 min and to obtain PPP, the blood was centrifuged at 1580× g for 10 min. Gel-filtered platelets were prepared using a method described previously [30,31]. Briefly, PRP was loaded to a column filled with sepharose gel, before the eluates were collected. The platelet concentration was adjusted to 2.5 × 10^8^ platelets/mL by using Tyrode's buffer.

### 5.5. Platelet Aggregation Assay

In vitro platelet aggregation was tested using a four-channel aggregometer (LBY-NJ4, Pulisheng Instrument Co. Ltd., Beijing, China) as previously described [32,33]. Gel-filtered human platelets were preincubated with the samples or vehicles for 5 min at 37 °C. After this, platelet aggregation was induced by ADP (20 µM), ristocetin (0.5 mg/mL), AYPGKF-NH_2_ (100 µM), SFLLRN-NH_2_ (2 µM), collagen (4 µg/mL), or thrombin (0.1 U/mL). The maximum aggregation rate was measured within 5 min with continuous stirring. The light transmittance was calibrated with Tyrode's buffer.

### 5.6. Arterio-Venous Shunt Model in Rats

An arterio-venous shunt model described previously [34,35] was used to estimate the effect of samples on thrombus formation. Briefly, SD rats were randomly divided into five groups (six rats in each group). DAA-8 (1, 5, 25 mg/kg), aspirin (50 mg/kg) or vehicle were injected into rats through the tail veins. The formed thrombus was weighed after the silk was dried for 40 min at 60 °C.

### 5.7. Bleeding Time Assay

The bleeding time was measured using a method described previously [36]. Briefly, the ICR mice were randomly divided into five groups (10 mice in each group), while DAA-8 (2.5, 10, and 50 mg/kg), aspirin (50 mg/kg) or vehicle were administrated through the tail veins. After 15 min, the mice were anesthetized using 5% chloral hydrate, before a cut of 3 mm at the tail tip was made. The remaining tail was immersed immediately into 12 mL of saline at 37 °C. Bleeding time was recorded within 20 min.

### 5.8. Statistical Analysis

The data were analyzed by using GraphPad Prism 6 (GraphPad Software, Inc., La Jolla, CA, USA). Results are expressed as the mean ± standard deviation (SD) values. The statistical significance of a two-sample comparison was evaluated by using Student's *t*-test, while multiple-sample comparisons were evaluated using one-way ANOVA analysis tested by Dunnett’s. *p* < 0.05 was considered to be statistically significant.

## Supplementary Materials

The following are available online at http://www.mdpi.com/2072-6651/10/8/332/s1, Figure S1: Mass spectrum of DAA-8. The molecular mass of DAA-8 was determined using ESI-MS. It was shown that the m/z value of [M+H]^+^ was 1033.25 Da.

## References

[1] Goto S., Tomita A. (2013). Antithrombotic therapy for prevention of various thrombotic diseases. Drug Dev. Res..

[2] Fredenburgh J.C., Gross P.L., Weitz J.I. (2017). Emerging anticoagulant strategies. Blood.

[3] Jackson S.P. (2007). The growing complexity of platelet aggregation. Blood.

[4] Stegner D., Nieswandt B. (2011). Platelet receptor signaling in thrombus formation. J. Mol. Med..

[5] Qiao J., Arthur J.F., Gardiner E.E., Andrews R.K., Zeng L., Xu K. (2018). Regulation of platelet activation and thrombus formation by reactive oxygen species. Redox Biol..

[6] Hao H.Z., He A.D., Wang D.C., Yin Z., Zhou Y.J., Liu G., Liang M.L., Da X.W., Yao G.Q., Xie W. (2015). Antiplatelet activity of loureirin A by attenuating Akt phosphorylation: In vitro studies. Eur. J. Pharm..

[7] Utkin Y.N. (2015). Animal venom studies: Current benefits and future developments. World J. Biol. Chem..

[8] Valenta J., Stach Z., Michalek P. (2015). Envenoming by crotalid snake chinese moccasin agkistrodonacutus bite—A case report. Prague Med. Rep..

[9] Li Q.B., Yu Q.S., Huang G.W., Tokeshi Y., Nakamura M., Kinjoh K., Kosugi T. (2000). Hemostatic disturbances observed in patients with snakebite in south China. Toxicon.

[10] Sajevic T., Leonardi A., Krizaj I. (2011). Hemostatically active proteins in snake venoms. Toxicon.

[11] Liu C.C., Lin C.C., Hsiao Y.C., Wang P.J., Yu J.S. (2018). Proteomic characterization of six Taiwanese snake venoms: Identification of species-specific proteins and development of a siscapa-mrm assay for cobra venom factors. J. Proteomics.

[12] Markland F.S. (1998). Snake venoms and the hemostatic system. Toxicon.

[13] Hutton R.A., Warrell D.A. (1993). Action of snake venom components on the hemostatic system. Blood Rev..

[14] Eble J.A., Niland S., Bracht T., Mormann M., Peter-Katalinic J., Pohlentz G., Stetefeld J. (2009). The α2β1 integrin-specific antagonist rhodocetin is a cruciform, heterotetrameric molecule. FASEB J..

[15] Navdaev A., Lochnit G., Eble J.A. (2011). The rhodocetinalphabeta subunit targets GPIb and inhibits von willebrand factor induced platelet activation. Toxicon.

[16] Fukuda K., Mizuno H., Atoda H., Morita T. (1999). Crystallization and preliminary X-ray studies of flavocetin-A, a platelet glycoprotein Ib-binding protein from the habu snake venom. Acta Crystallogr. D. Biol. Crystallogr..

[17] Arlinghaus F.T., Eble J.A. (2013). The collagen-binding integrin α2β1 is a novel interaction partner of the *Trimeresurus flavoviridis* venom protein flavocetin-A. J. Biol. Chem..

[18] Hsu C.C., Wu W.B., Huang T.F. (2008). A snake venom metalloproteinase, kistomin, cleaves platelet glycoprotein VI and impairs platelet functions. J. Thromb. Hemost..

[19] Samah S., Fatah C., Jean-Marc B., Safia K.T., Fatima L.D. (2017). Purification and characterization of cc-lec, c-type lactose-binding lectin: A platelet aggregation and blood-clotting inhibitor from cerastescerastes venom. Int. J. Biol. Macromol..

[20] Yoshida N., Yoshikawa T. (2008). Basic and translational research on proteinase-activated receptors: Implication of proteinase/proteinase-activated receptor in gastrointestinal inflammation. J. Pharm. Sci..

[21] Font J., Simeon M., Simard C., Allouche S., Plane A.F., Ferchaud V., Brionne M., Rouet R., Nowoczyn M., Manrique A. (2018). PAR1 contribution in acute electrophysiological properties of oral anticoagulants in rabbit pulmonary vein sleeve preparations. Fundam. Clin. Pharm..

[22] Talati N., Kamato D., Piva T.J., Little P.J., Osman N. (2018). Thrombin promotes PAI-1 expression and migration in keratinocytes via ERK dependent Smad linker region phosphorylation. Cell. Signal..

[23] Matsuno H., Ishisaki A., Nakajima K., Kato K., Kozawa O. (2010). A peptide isolated from αB-crystallin, which is a novel and potent inhibitor of platelet aggregation via dual prevention of PAR-1 and GPIb/V/IX. J. Thromb. Hemost..

[24] Tawfik B., Pollard E., Shen Y.M. (2018). The novel protease-activated receptor 1 antagonist vorapaxar as a treatment for thrombosis in afibrinogenemia. Semin. Thromb. Hemost..

[25] Baker N.C., Lipinski M.J., Lhermusier T., Waksman R. (2014). Overview of the 2014 food and drug administration cardiovascular and renal drugs advisory committee meeting about vorapaxar. Circulation.

[26] Wang W.J., Shih C.H., Huang T.F. (2005). Primary structure and antiplatelet mechanism of a snake venom metalloproteinase, acurhagin, from agkistrodonacutus venom. Biochimie.

[27] Zhu Z., Gao Y., Zhu Z., Yu Y., Zhang X., Zang J., Teng M., Niu L. (2009). Structural basis of the autolysis of Aahiv suggests a novel target recognizing model for ADAM/reprolysin family proteins. Biochem. Biophys. Res. Commun..

[28] Liang X.X., Chen J.S., Zhou Y.N., Qiu P.X., Yan G.M. (2001). Purification and biochemical characterization of F II_a_, a fibrinolytic enzyme from *Agkistrodon acutus* venom. Toxicon.

[29] Wang R., Qiu P., Jiang W., Cai X., Ou Y., Su X., Cai J., Chen J., Yin W., Yan G. (2008). Recombinant fibrinogenase from *Agkistrodon acutus* venom protects against sepsis via direct degradation of fibrin and TNF-α. Biochem. Pharm..

[30] Yi W., Li Q., Shen J., Ren L., Liu X., Wang Q., He S., Wu Q., Hu H., Mao X. (2014). Modulation of platelet activation and thrombus formation using a Pan-PI3K inhibitor S14161. PLoS ONE.

[31] Li W., Tang X., Yi W., Li Q., Ren L., Liu X., Chu C., Ozaki Y., Zhang J., Zhu L. (2013). Glaucocalyxin A inhibits platelet activation and thrombus formation preferentially via GPVI signaling pathway. PLoS ONE.

[32] Maione F., De Feo V., Caiazzo E., De Martino L., Cicala C., Mascolo N. (2014). Tanshinone IIA, a major component of *Salvia milthorriza* bunge, inhibits platelet activation via erk-2 signaling pathway. J. Ethnopharm..

[33] Geraghty D.P., Ahuja K.D., Pittaway J., Shing C., Jacobson G.A., Jager N., Jurkovic S., Narkowicz C., Saunders C.I., Ball M. (2011). In vitro antioxidant, antiplatelet and anti-inflammatory activity of *Carpobrotusrossii* (pigface) extract. J. Ethnopharm..

[34] Chen M., Ye X., Ming X., Chen Y., Wang Y., Su X., Su W., Kong Y. (2015). A novel direct factor Xa inhibitory peptide with anti-platelet aggregation activity from agkistrodonacutus venom hydrolysates. Sci. Rep..

[35] Sperzel M., Huetter J. (2007). Evaluation of aprotinin and tranexamic acid in different in vitro and in vivo models of fibrinolysis, coagulation and thrombus formation. J. Thromb. Hemost..

[36] Sugidashi A., Asai F., Ogama T., Inoue T., Koike H. (2000). The in vivo pharmacological profile of CS-747, anovel antiplatelet agent with platelet ADP receptor antagonist properties. Br. J. Pharm..

