# Supplementary material for: Purification and Characterization of a Novel Antiplatelet Peptide from Deinagkistrodon acutus Venom
Source: Toxins (Basel). 2018 Aug 16;10(8):332. doi: 10.3390/toxins10080332 (PMC6115707; doi:10.3390/toxins10080332)
Supplement: Supplementary file 1 [file toxins-10-00332-s001.pdf]

Supplementary Material: Purification and Characterization of a NovelAntiplatelet Peptide from *Deinagkistrodon acutus* Venom

Yi Kong, Qing Sun, Qi Zhao and Yaqiong Zhang

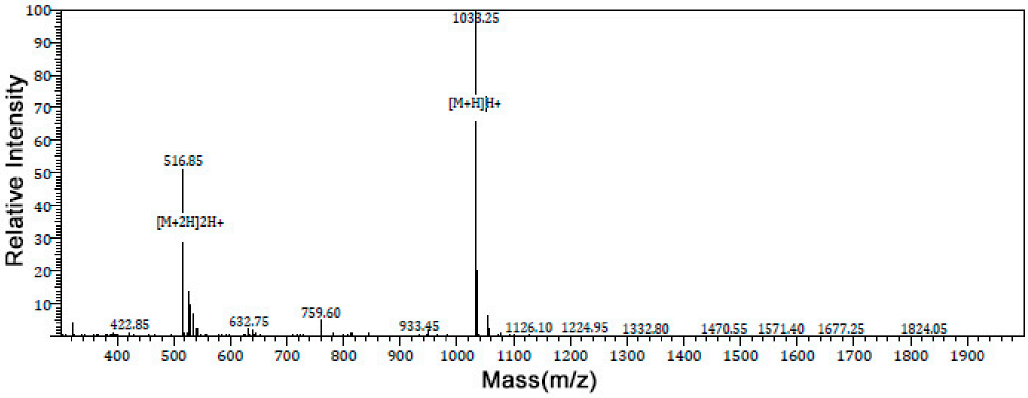

Figure S1. Mass spectrum of DAA-8. The molecular mass of DAA-8 was determined using ESI-MS. It was shown that the *m*/*z* value of $[M+H]^+$ was 1033.25 Da.
